# Cross-Stress Adaptation in a Piezophilic and Hyperthermophilic Archaeon From Deep Sea Hydrothermal Vent

**DOI:** 10.3389/fmicb.2020.02081

**Published:** 2020-09-10

**Authors:** Weishu Zhao, Xiaopan Ma, Xiaoxia Liu, Huahua Jian, Yu Zhang, Xiang Xiao

**Affiliations:** ^1^State Key Laboratory of Microbial Metabolism, School of Life Sciences and Biotechnology, Shanghai Jiao Tong University, Shanghai, China; ^2^State Key Laboratory of Ocean Engineering, Shanghai Jiao Tong University, Shanghai, China; ^3^School of Life Sciences and Biotechnology, Shanghai Jiao Tong University, Shanghai, China

**Keywords:** extreme adaptation, cross-stress adaptation, hyperthermophile, piezophile, archaea, extremophile, proteomic analysis

## Abstract

Hyperthermophiles, living in environments above 80°C and usually coupling with multi-extreme environmental stresses, have drawn great attention due to their application potential in biotechnology and being the primitive extant forms of life. Studies on their survival and adaptation mechanisms have extended our understanding on how lives thrive under extreme conditions. During these studies, the “cross-stress” behavior in various organisms has been observed between the extreme high temperature and other environmental stresses. Despite the broad observation, the global view of the cross-stress behavior remains unclear in hyperthermophiles, leaving a knowledge gap in our understanding of extreme adaptation. In this study, we performed a global quantitative proteomic analysis under extreme temperatures, pH, hydrostatic pressure (HP), and salinity on an archaeal strain, *Thermococcus eurythermalis* A501, which has outstanding growth capability on a wide range of temperatures (50–100°C), pH (4–9), and HPs (0.1–70 MPa), but a narrow range of NaCl (1.0–5.0 %, w/v). The proteomic analysis (79.8% genome coverage) demonstrated that approximately 61.5% of the significant differentially expressed proteins (DEPs) responded to multiple stresses. The responses to most of the tested stresses were closely correlated, except the responses to high salinity and low temperature. The top three enriched universal responding processes include the biosynthesis and protection of macromolecules, biosynthesis and metabolism of amino acids, ion transport, and binding activities. In addition, this study also revealed that the specific dual-stress responding processes, such as the membrane lipids for both cold and HP stresses and the signal transduction for both hyperosmotic and heat stresses, as well as the sodium-dependent energetic processes might be the limiting factor of the growth range in salinity. The present study is the first to examine the global cross-stress responses in a piezophilic hyperthermophile at the proteomic level. Our findings provide direct evidences of the cross-stress adaptation strategy (33.5% of coding-genes) to multiple stresses and highlight the specific and unique responding processes (0.22–0.63% of coding genes for each) to extreme temperature, pH, salinity, and pressure, which are highly relevant to the fields of evolutionary biology as well as next generation industrial biotechnology (NGIB).

## Introduction

Cross-stress adaptation, a phenomenon that a given stress confers a fitness advantage (or disadvantage) to other unrelated stresses, has been observed in many organisms across the tree of life, ranging from microbes to plants and humans (Boussiba et al., 1975; Dragosits et al., 2013; Chauhan et al., 2015; Zorraquino et al., 2016). In the past three decades, researchers reported many special genes responding to a certain stress; however, more and more studies found that these responding genes could respond to more than one stress at the same time. The cellular machinery and metabolism of individuals could be flexible to cope with a certain degree of environmental volatility. The cross-stress adaptation in extremophiles, who are living in such harsh environments and facing multiple stresses, attracted a lot of interests. To cope with the multiple extreme conditions, the biomolecular adaptations should be unique and wide ranging. Studies on their survival and adaptation mechanisms extended our understanding on how lives thrive under extreme conditions (Ambily Nath and Loka Bharathi, 2011; Zhang et al., 2015). Although various environmental stresses, including temperature, pressure, pH, oxidative stress, radiation, drought, heavy metals, and toxins, have been investigated in many different extremophilic strains (Ambily Nath and Loka Bharathi, 2011; Zhang et al., 2015), the genetic basis of multi-extreme adaptation has not been systematically elucidated in extremophiles.

Deep sea hydrothermal vents are one of the most multi-extreme environments on modern earth and have similar conditions to those on early earth (Jørgensen and Boetius, 2007). *Thermococcales*, a group of organotrophic anaerobic hyperthermophilic archaea, are widely distributed and predominated in the deep sea hydrothermal vents with a great biomass up to 10^7^ cells/g sediment sample (Takai and Nakamura, 2011). *Thermococcales* are one of the well-adapted orders to the dramatic environmental fluctuations in physical and chemical factors of hydrothermal vents during a long history of the earth, which was reported as the only active group in the sediments that are 1,626 m below the sea floor and 111 million years old (Roussel et al., 2008). They are generally observed to grow over a wide temperature range, usually wider than 40°C of difference, but with a concise genome (1.7–2.3 Mbp) (Zhao and Xiao, 2017). At the same time, they are in a very rare archaea group with piezophiles. Due to the small genome size and deeply branching phylogenetic status, *Thermococcales* have relatively simple metabolic pathways, which make them ideal organisms for researches on multi-extreme adaptation. Current studies of *Thermococcales* adaptation to various extreme environments, such as heat, cold, high hydrostatic pressure (HP), and hyperosmotic and oxidative stresses, have identified many stress responding genes and proteins. These stress responses include heat shock proteins (Shockley et al., 2003; Cario et al., 2016), compatible solutes (Müller et al., 2005; Empadinhas and da Costa, 2006), membrane lipid components (Lattuati et al., 1998; Meador et al., 2014; Cario et al., 2015a), amino acid requirements (Cario et al., 2015b), etc. Many of the above responding genes and proteins were reported in more than one stress responses in different strains, suggesting the possibility of universal adaptation strategies to cope with multi-extreme adaptation (Jia et al., 2015; Zhang et al., 2015). However, a global view of the cross-stress responses is still lacking.

A robust strain with stable and rapid growth under changing conditions could provide an ideal platform for both the study of adaptation mechanisms and the development of next generation industrial biotechnology (NGIB). As platform strains, the wide-ranging strains with multi-stress adaptation would be even better than those with narrow range or adapted to single stress (Chen and Jiang, 2018). In general, most organisms show growth over a range of 25–40°C and 2–3 pH units at atmospheric pressure (Madigan et al., 2015). However, some extremophilic archaea like *Thermococcales* could live beyond the canonical growth range. *Thermococcus eurythermalis* A501 is one such example, which can grow within a range over 50°C (50–100°C), 5 pH units (4.0–9.0), and 700 barometric pressure (0.1–70 MPa), although with a narrow range on NaCl (1.0–5.0 %, w/v) (Zhao et al., 2015). Indeed, the growth capability of *T. eurythermalis* A501 in terms of temperature and pH range is extraordinary among both normal and extremophilic microbes (Supplementary Figure S1).

In this study, *T. eurythermalis* A501 was selected to investigate the cross-stress mechanism of microbial response to various stresses on temperature, pH, NaCl concentration, and pressure. Global proteomic analysis with a high coverage of genome-predicted proteins was performed to be accessible to discover the responding processes that were controlled by both transcriptional as well as post-transcriptional mechanisms. The testing environmental stresses were determined by two criteria: biomass sufficient for harvest (more than 5 × 10^7^ cells/ml) and proximity to a growth boundary (Table 1 and Supplementary Figure S2). The optimal growth condition (85°C, pH 7.0, 2.3% NaCl, 0.1 MPa or 10 MPa) was used as a control to identify the significant differentially expressed proteins (DEPs). Statistical analysis based on DEPs revealed the correlations among responses of different stresses. The Gene Ontology (GO) functional enrichment of DEPs under each stress condition identified universal responses to multi-stresses, specific responses to dual stresses, and unique responses to each tested stress.

**TABLE 1:** Summary of growth profiles and differential expressed proteins (DEPs) in quantitative proteomic analysis of *Thermococcus eurythermalis* A501.

Condition	Growth profiles		Number of DEPs		
	Growth rate (h^–1^)	Max yield (10^8^ cells/ml)	Up-expressed	Down-expressed	Unique
HpH	0.93 ± 0.01	2.89 ± 0.01	108	68	17
LpH	0.57 ± 0.01	1.82 ± 0.09	64	135	21
HS	0.78 ± 0.01	0.75 ± 0.02	324	133	66
LS	0.98 ± 0.01	1.69 ± 0.01	222	76	34
HT	0.94 ± 0.13	0.92 ± 0.01	120	193	66
LT	0.40 ± 0.02	0.75 ± 0.01	158	252	82
HP	0.91 ± 0.13	1.43 ± 0.01	135	128	25
HT@10 MPa	1.20 ± 0.07	1.75 ± 0.38	233	223	NC
HT@40 MPa	0.67 ± 0.04	1.01 ± 0.29	193	203	NC
HP@95°C	0.67 ± 0.04	1.01 ± 0.29	139	162	NC
Opt	1.27 ± 0.04	2.83 ± 0.05	–	–	–

*DEPs were defined as proteins whose expressions were up-expressed > 1.2 or down-expressed < 0.8 with a p-value < 0.05 by using the samples under the optimal condition (Opt) as a reference. Standard deviations were calculated on the quadruplicates under each condition. Unique DEPs were defined as those differentially expressed under single stress condition. NC, not counted. –, used as control.*

## Materials and Methods

### Cell Culture of *T. eurythermalis* Under Different Environmental Stresses

*Thermococcales eurythermalis* A501 was cultured in medium under different culture conditions as previously described (Zhao et al., 2015). It was inoculated into 50 ml of *Thermococcales* rich medium (TRM) in anaerobic serum bottles with N_2_ as the gas phase at atmospheric pressure, and 50-ml syringes with TRM were used at HP (Zhao et al., 2015). The environmental stresses for cultivation were low temperature (65°C), high temperature (95°C), low pH (pH 4), high pH (pH 9), low salinity (1.5% NaCl, w/v), high salinity (4.5% NaCl, w/v), and HP (40 MPa), and the optimal culture condition (85°C, pH 7, 2.3% NaCl, 0.1 MPa or 10 MPa) was used as the control. The media were inoculated with approximately 10^6^ cells ml^–1^ and the cells were grown to mid-exponential phase according to the growth curve (Supplementary Figure S2).

### Cell Harvest and Protein Extraction

*Thermococcus eurythermalis* A501 was cultured separately in quadruplicates for each condition, and cells were harvested as described by Moon et al. (2012). The protein extraction was performed with a similar method to that used for the thermophilic bacteria *Thermoanaerobacter tengcongensis* (Chen et al., 2013). The collected cells were suspended in lysis buffer (7 M urea, 2 M thiourea, 4% CHAPS, 40 mM Tris–HCl, pH 8.5, 1 mM PMSF, and 2 mM EDTA) and sonicated. The proteins were reduced with 10 mM dithiothreitol and then alkylated by 55 mM iodoacetamide. The treated protein mixtures were precipitated by adding fourfold volume of chilled acetone. After centrifugation, the pellet was dissolved in 500 M tetraethylammonium bicarbonate (TEAB, pH 8.5) and sonicated on ice. After centrifugation, the proteins in the supernatant were stored at −80°C for further analysis.

### Proteomic Analysis

iTRAQ analysis was performed at BGI (Shenzhen, China). The mass spectrometry proteomics data have been deposited to the ProteomeXchange Consortium via the PRIDE partner repository with the dataset identifier PXD018974 (for pH data), PXD018991 (for temperature data), PXD018994 (for NaCl data), and PXD019007 (for HP data) (Perez-Riverol et al., 2019).

#### iTRAQ Labeling and SCX Fractionation

Total protein (100 μg) was taken from each sample solution, and then the protein was digested with Trypsin Gold (Promega, Madison, WI, United States) at a protein:trypsin ratio of 20:1 at 37°C for 12 h. The protein concentrations were determined by the Bradford method. After trypsin digestion, peptides were dried by vacuum centrifugation. Peptides were reconstituted in 0.5 M TEAB and processed according to the manufacturer’s protocol for 8-plex iTRAQ reagent (Applied Biosystems, Foster City, CA, United States). The labeled peptide mixtures were then pooled and dried by vacuum centrifugation. SCX chromatography was performed with an LC-20AB HPLC Pump system (Shimadzu, Kyoto, Japan). The iTRAQ-labeled peptide mixtures were reconstituted with 4 ml of buffer A (25 mM NaH_2_PO_4_ in 25% v/v acetonitrile, pH 2.7) and loaded onto a 4.6 mm × 250 mm Ultremex SCX column containing 5-μm particles (Phenomenex, Torrance, CA, United States). The peptides were eluted at a flow rate of 1 ml/min with a gradient of buffer A for 10 min, 5–60% buffer B (25 mM NaH_2_PO_4_ and 1 M KCl in 25% v/v acetonitrile, pH 2.7) for 27 min, and 60–100% buffer B for 1 min. The system was then maintained at 100% buffer B for 1 min before equilibrating with buffer A for 10 min prior to the next injection. Elution was monitored by measuring the absorbance at 214 nm, and fractions were collected every 1 min. The eluted peptides were pooled into 20 fractions, desalted with a Strata XC18 column (Phenomenex, Torrance, CA, United States), and vacuum-dried.

#### LC-MS/MS Analysis

The supernatant was separated using an LC-20AD Nano-HPLC system (Shimadzu, Kyoto, Japan). Each fraction was resuspended in buffer I (5% acetonitrile and 0.1% formic acid) and centrifuged at 20,000 × *g* for 10 min; the final concentration of peptide was approximately 0.5 μg/μl. Then, 10 μl of supernatant was loaded on the LC-20AD nano-HPLC by the autosampler onto a 2-cm C18 trap column. The peptides were eluted onto a 10-cm analytical C18 column (inner diameter, 75 μm) packed in-house. The samples were loaded at 8 μl/min for 4 min, and the 35-min gradient was run at 0.3 μl/min starting from 2 to 35% buffer II (95% acetonitrile and 0.1% formic acid), followed by a 5-min linear gradient to 60%. Then, there was a 2-min linear gradient to 80%, maintenance at 80% buffer II for 4 min, and a return to 5% in 1 min.

Data acquisition was performed with a TripleTOF 5600 System (AB SCIEX, Concord, ON, United States) fitted with a Nanospray III source (AB SCIEX, Concord, ON, United States) and a pulled quartz tip as the emitter (New Objectives, Woburn, MA, United States). Data were acquired using an ion spray voltage of 2.5 kV, a curtain gas of 30 psi, a nebulizer gas of 15 psi, and an interface heater temperature of 150°C. The MS was operated with an RP of greater than or equal to 30,000 FWHM for TOF MS scans. For IDA, survey scans were acquired in 250 ms, and as many as 30 product ion scans were collected if exceeding a threshold of 120 counts per second (counts/s) and with a 2+ to 5+ charge state. Total cycle time was fixed to 3.3 s. The Q2 transmission window was 100 Da for 100%. Four time bins were summed for each scan at a pulser frequency value of 11 kHz through monitoring of the 40-GHz multi-channel TDC detector with four-anode channel detect ion. A sweeping collision energy setting of 35 ± 5 eV coupled with iTRAQ adjusted rolling collision energy was applied to all precursor ions for collision-induced dissociation. Dynamic exclusion was set for 1/2 of peak width (15 s), and then the precursor was refreshed off the exclusion list.

#### Protein Identification and Quantitative Data Analysis

Raw MS/MS data files acquired from the Orbitrap were converted into MGF files by Proteome Discoverer 1.2 (Thermo Fisher Scientific, Waltham, MA, United States). Protein identification was performed by using MGF files identified by the Mascot search engine (Matrix Science, London, United Kingdom; version 2.3.02) against a database containing 2181 predicted proteins in *T. eurythermalis* A501 (The GenBank/EMBL/DDBJ accession number: CP008887-CP008888) (Zhao and Xiao, 2015), as well as the public database (NCBInr, SwissProt, and UniProt). For protein identification, a mass tolerance of 0.05 Da (ppm) was permitted for intact peptide masses and 0.1 Da for fragmented ions, with allowance for one missed cleavage in the trypsin digests. Gln-pyro-Glu (N-term Q), oxidation (M), and iTRAQ8plex (Y) were used as the potential variable modifications, and carbamidomethyl (C), iTRAQ8plex (N-term), and iTRAQ8plex (K) were used as fixed modifications. The charge states of peptides were set to +2 and +3. Specifically, an automatic decoy database search was performed in Mascot by choosing the decoy checkbox in which a random sequence from the database is generated and tested for raw spectra as well as the real database. To reduce the probability of false peptide identification, only peptides with significance scores (≥ 20) at the 99% confidence interval by a Mascot probability analysis greater than “identity” were counted as identified. A unique protein with at least two unique peptides was qualified whose false discovery rate (FDR) < 0.01. For protein quantitation, a protein had to contain at least two unique peptides. The quantitative protein ratios were weighted and normalized by the median ratio in Mascot (Chen et al., 2013).

### Statistical Analysis

*p*-values were calculated using a paired *t*-test. Proteins with *p*-values less than 0.05 and fold changes higher than 1.2 were considered significant and treated as DEPs and then log_2_-transformed for further analysis in heatmaps, PCA, and correlation. Heatmaps were generated using “gplots” package in R and clustered and reordered by both rows and columns using Euclidean distance and complete linkage clustering method; PCA was conducted by “vegan” package in R; the correlation matrix was calculated by Spearman’s method in the function “cor” in R.

The significant responding processes to each stress were identified by mapping the DEPs into the GO database^1^, and then, hypergeometric tests were performed to eliminate the influence of stochastic background processes. The molecular function and biological process terms of the GO database with *p*-values < 0.05 under each stress were defined as the significantly enriched GO terms, which were used to generate a profile of crucial responding processes under each stress.

## Results

### Global Proteomic Analysis Under Various Stress Conditions

Global proteomic data with a high genome coverage were obtained by iTRAQ-based proteomic analysis (see section “Materials and Methods”). Cells under each tested condition were performed separately in quadruplicates. The responses of *T. eurythermalis* A501 to extreme pH (acid or alkaline, denoted LpH or HpH), temperature (cold or heat, denoted LT and HT), salinity (hypo- or hyperosmotic, denoted LS and HS), and HP were investigated using differential proteomic analysis. A total of 1740 proteins were identified by the Mascot search engine (Matrix Science, London, United Kingdom; version 2.3.02), corresponding to 79.8% of the 2181 protein-encoding genes predicted in the *T. eurythermalis* A501 complete genome (Zhao and Xiao, 2015), including 1556 distinct proteins (71.3% of encoding genes) with more than two peptide fragments identified at least twice across all experiments. These 1556 proteins were then used for subsequent differential expression analysis. The samples that were cultivated under optimal conditions were used as a reference to identify the differential expression. We defined DEPs as those up-expressed > 1.2 or down-expressed < 0.8 with a *p-*value < 0.05 (*t*-test, multiple testing correction) under each stress condition, which is the cutoff commonly used for iTRAQ-based proteomics analysis of both prokaryotes and eukaryotes (Wang et al., 2016, 2020; Cheng et al., 2017; Hou et al., 2019; Li et al., 2019). The details of the specific growth rates, maximum growth yields, and number of DEPs under each condition are presented in Table 1.

**FIGURE 1:**
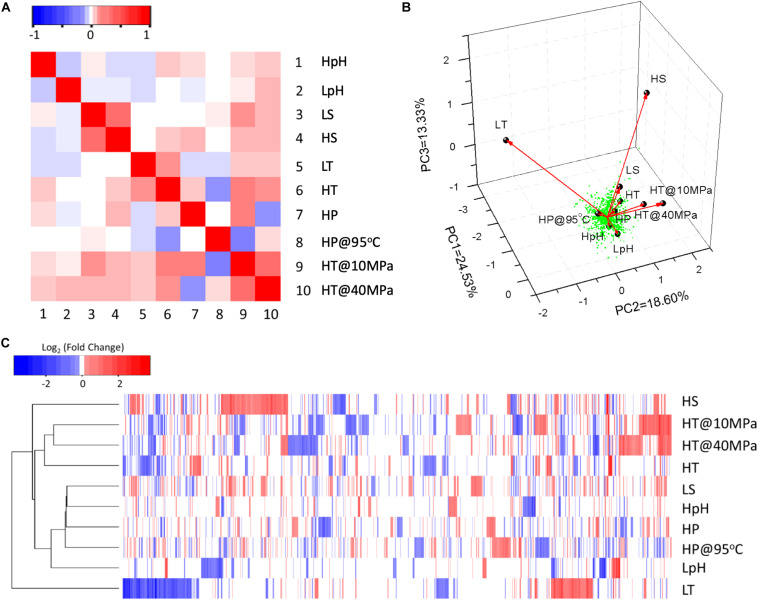
The statistical analysis of DEPs responding to different environmental stresses. **(A)** Correlation matrix of different stress responses based on DEPs. Spearman’s method was used to calculate the matrix with the “cor” function in the R program. Matrix elements have been scaled so that the smallest negative element is –1, the largest positive element is +1, and all elements retain their sign. **(B)** Three-dimensional plots of PCA of DEPs. The type of stress culture condition was used as the environmental variable. Black dots and red arrows present the strength and direction of the effects of environmental variables. The green dots indicate each significant DEP. **(C)** Clustering heatmap of significant DEPs. Up- and down-expressed proteins are shown in red and blue, respectively. Raw data of whole proteomic data were log_2_-transformed and then clustered and reordered by both rows and columns using the Euclidean distance method and complete linkage cluster method. Symbols of tested conditions are as follows: “HpH,” pH 8.8 vs. pH 7.0; “LpH,” pH 4.4 vs. pH 7.0; “LS,” 1.5% NaCl vs. 2.3% NaCl; “HS,” 4.5% NaCl vs. 2.3% NaCl; “LT,” 65°C vs. 85°C; “HT,” 95°C vs. 85°C; “HP,” 85°C at 40 MPa vs. 85°C at 10 MPa; “HP@95°C,” 40 MPa at 95°C vs. 10 MPa at 95°C; “HT@10 MPa,” 95°C at 10 MPa vs. 85°C at 10 MPa; “HT@40 MPa,” 95°C at 40 MPa vs. 85°C at 40 MPa.

The statistical analysis of DEPs (see section “Materials and Methods”) revealed the correlation of responses among various stresses. According to the correlation matrix (Figure 1A), positive correlations were observed between the responses to LS and HS, LT and HT, whereas a negative correlation was observed between the responses to LpH and HpH. The response to HP was positively correlated with the responses to HpH, HS, and HT, while it was negatively correlated with the responses to LpH and LT. This result indicates that the adaptation to pressure is globally related to other stress adaptations. Interestingly, both the clustering (Figure 1B) and PCA plot (Figure 1C) presented that the DEPs responding to LT and HP stresses were definitely distinctive from DEPs responding to other stresses.

**FIGURE 2:**
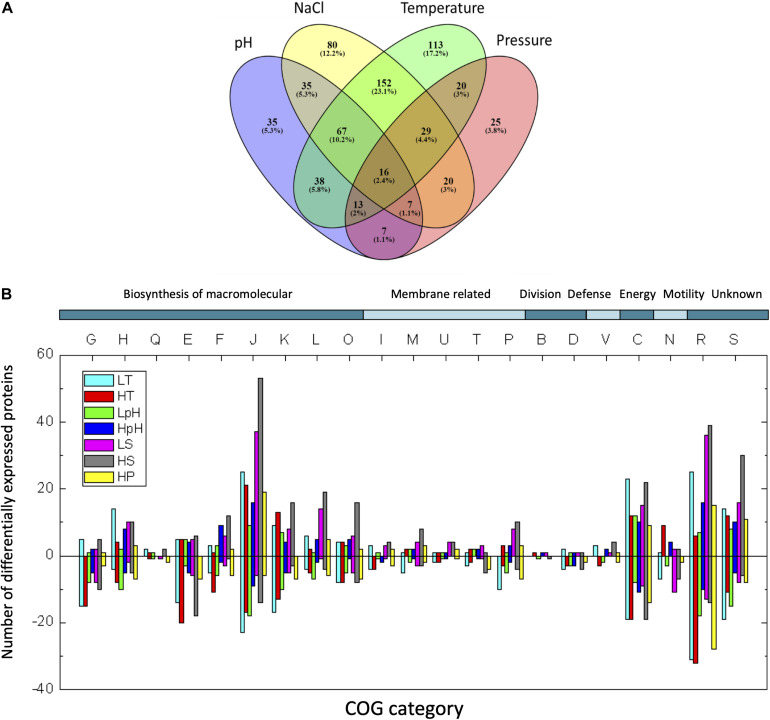
**(A)** Venn diagram of DEP numbers under different stress conditions. Temperature, union of significant DEPs at both low and high temperature stress; NaCl, union of significant DEPs under LS and HS; pH, union of significant DEPs under LpH and HpH; Pressure, significant DEPs at HP stress. Percent of the total DEPs in all tested stresses is shown in parenthesis. **(B)** COG classification of DEPs. The following COG categories were included: G, carbohydrate transport and metabolism; H, coenzyme transport and metabolism; Q, secondary metabolite biosynthesis, transport, and catabolism; E, amino acid transport and metabolism; F, nucleotide transport and metabolism; J, translation, ribosomal structure, and biogenesis; K, transcription; L, replication, recombination, and repair; O, post-translational modification, protein turnover, and chaperones; I, lipid transport and metabolism; M, cell wall/membrane/envelope biogenesis; U, intracellular trafficking, secretion, and vesicular transport; T, signal transduction mechanisms; P, inorganic ion transport and metabolism; B, chromatin structure and dynamics; D, cell cycle control, cell division, and chromosome partitioning; V, defense mechanisms; C, energy production and conversion; N, cell motility; R, general function prediction only; S, function unknown. The numbers of up- or down-regulated proteins are indicated as bars with positive or negative numbers on the histogram, respectively.

We designated the common DEPs and unique DEPs from the total DEPs obtained under all tested conditions (Supplementary Figure 2A and Supplementary Data S1). Approximately 61.5% of the total DEPs respond to three or more types of stresses among temperature, pH, salinity, and pressure, indicating the common responses to multiple stresses in *T. eurythermalis* A501. In contrast to the common DEPs, the unique DEPs to each stress are designated as those that only responded to a single stress without cross-stress behavior to other stresses. Only 1.5–7% of the total DEPs are unique responding to only one stress (Figure 2). The number of unique DEPs for each stress is presented in Table 1.

### Metabolic Function Predictions for DEPs Under Each Stress

Differentially expressed proteins were mapped to the complete genome to obtain gene expression profiles under each stress. The metabolic function of DEPs was predicted through the following three steps: (i) The functional groups of DEPs were clarified by using the Cluster of Orthologous Groups database (COG) (Figure 2B). (ii) To further specify the pathways in *T. eurythermalis* A501, the *Thermococcales*-specific pathways in substance metabolism and energy metabolism were manually constructed according to the sequence similarity with significant homology (>30% identity) to the identified or predicted proteins in the related *Thermococcales* strains, such as *Thermococcus kodakarensis* KOD1 (Fukui et al., 2005; Sato et al., 2007; Nohara et al., 2014) and *Pyrococcus furiosus* COM1 (Pisa et al., 2007; Schut et al., 2007; Strand et al., 2010) (Supplementary Figure S3 and Supplementary Data S2). (iii) To highlight the significant responding processes to each stress, we mapped the DEPs into the GO database, and then, hypergeometric tests were performed to analyze the GO terms, which could eliminate the influence of stochastic background processes. The molecular function and biological process terms of the GO database with *p*-values < 0.05 under each stress were defined as the significantly enriched GO terms, which were used to generate a profile of crucial responding processes under each stress (Supplementary Datas S3, S4).

In total, 732 DEPs were involved in the enriched universal responding processes, constituting 33.5% of the predicted protein-encoding genes in the complete genome.

### Universal Responding Processes for Cross-Stress Adaptation

**FIGURE 3:**
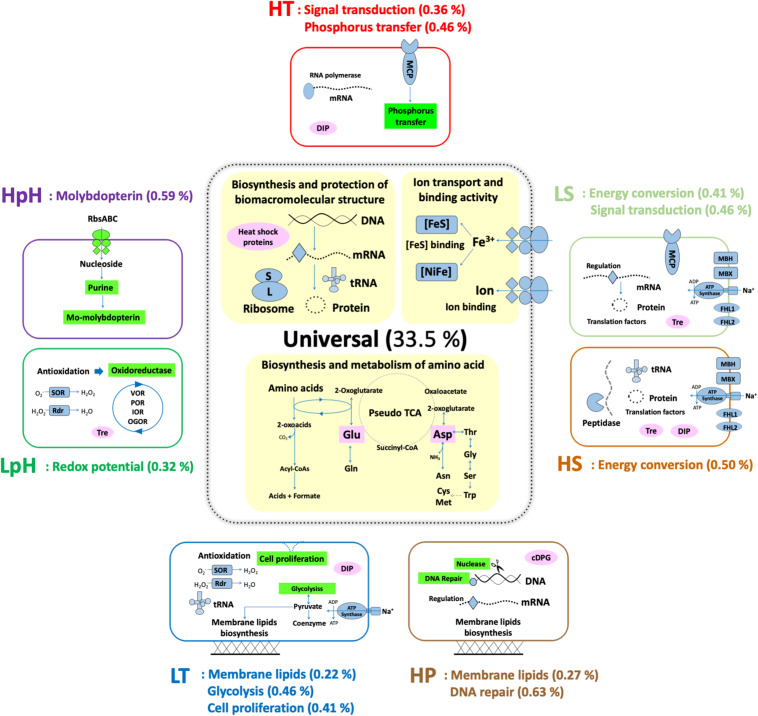
Proposed molecular functions and biological processes of *T. eurythermalis* A501 responding to different stresses. All the presented functions and processes were enriched with *p*-values less than 0.05. Universal responding processes are shown with a yellow background. The unique responses to each stress have a light green background. The compatible solutes and chaperonin are in pink. Presented in brackets is the number of DEPs in enriched pathways under each condition versus the all predicted protein-encoding genes in the complete genome of *T. eurythermalis* A501. MCP, methyl-accepting chemotaxis protein; MBH, membrane-bound hydrogenase; MBX, membrane-bound complex; FHL1 and FHL2, formate hydrogen lyase complexes 1 and 2; SOR, superoxide reductase; Rdr, rubrerythrin; VOR, ketoisovalerate oxidoreductase; POR, pyruvate oxidoreductase; IOR, indolepyruvate oxidoreductase; OGOR, oxoglutarate oxidoreductase; DIP, di-myo-inositol phosphate; Tre, Trehalose; cDPG: cyclic 2,3-diphosphoglycerate.

According to the pathway enrichment results, the following three processes were the major universal responding processes, which were significantly enriched under most tested stresses: (i) biosynthesis and protection of macromolecules, (ii) biosynthesis and metabolism of amino acids, and (iii) ion transporting and binding (Figure 3 and Supplementary Data S3). Among these three, the former two have been reported in various strains as responding processes to the stresses of HT, HS, HP, as well as oxidative stress (Table 2), which were expected to match the observation in *T. eurythermalis* A501 (additional data were presented in Supplementary Text and Supplementary Table S1), while the importance of ion transport and binding was beyond our expectations.

**TABLE 2:** Universal responding processes to multiple stresses in *T. eurythermalis* A501 and other hyperthermophilic archaea.

	Function	Pathway	HpH		LpH		LS		HS		LT		HT		HP		O_2_
			A	R	A	R	A	R	A	R	A	R	A	R	A	R	R
(I)	**Compatible solute**	DIP							+	[14]	+		+	[4][5][14]			[14]
		Trehalose			+		+		+					[2]		[1] [12]	
		cDPG													+	[17]	
		MG	−		−	‘	−		−		−		−	[3]	−		
		Glu and Asp	+		+	[9]	+		+	[14]	+	[15]	+	[4][14]	+	[12]	[14]
	**Information processing**	Purine/pyrimidine	+		+		+	[6]	+	[14]	+	[15]	+		+	[7] [12]	
		Ribose					+		+		+		+		+		
		Transcriptional regulator	+	[11]	+	[11]	+		+	[6] [14]	+	[15]	+	[14]	+	[7] [12]	[14]
		mRNA/rRNA/tRNA	+		+		+		+		+	[15]	+	[14]		[7]	
		Ribosome protein	+		+		+	[6]	+	[14]	+	[15]	+		+		
		DNA repair	+	[1]	+	[1]	+		+		+	[1]	+	[14]	+	[7]	[14]
		CRISPR	+				+		+		+			[2]	+	[12]	[8]
		Molybdopterin	+		+		+		+		+		+		+		[14]
	**Others**	CoA and MVA pathway	+		+		+		+	[6]	+	[15]	+	[14]		[7]	
		Propionate pathway	+		+		+		+		+	[15]	+		+		
		Chaperone proteins	+	[1]	+	[1]	+	[6]	+	[6]	+	[1][13]	+	[2][13] [14]	+	[7][12]	
(II)	**Amino acids**	AA biosynthesis	+		+		+	[6]	+	[6][14]	+	[15]	+	[2][14]	+	[7][12]	[8] [14]
		Protease/peptidase	+		+		+		+	[6] [14]	+		+	[2] [14]		[7]	[14]
		Peptide transporter			+		+	[6]	+	[6]	+		+		+	[7]	
		Glu transport	+		+	[9]			+		+		+				
		Proteasome				[9]			+		+		+	[2]			
(III)	**Ion**	Fe transporter	+		+		+		+	[6]	+		+		+	[7]	
		Other ion transporters		[10] [11]		[9] [11]		[6]		[6]		[1]				[7]	
		[FeS] cluster	+		[16]		+		+		+		+		+		
		[NiFe] hydrogenase	+		+		+		+		+						

*References mentioned above: [1] (Ambily Nath and Loka Bharathi, 2011); [2] (Shockley et al., 2003); [3] (Esteves et al., 2014); [4] (Borges et al., 2010); [5] (Keese et al., 2010); [6] (Coker et al., 2007); [7] (Vezzi et al., 2005); [8] (Strand et al., 2010); [9] (Lund et al., 2014); [10] (Padan et al., 2005); [11] (Krulwich et al., 2011); [12] (Vannier et al., 2015); [13] (Gao et al., 2015); [14] (Jia et al., 2015); [15] (Trauger et al., 2008); [16] (Berezovsky and Shakhnovich, 2005); [17] (Shima et al., 1998). A, significant DEPs were observed in A501 in this study; R, reported publications, the number of the related references is shown in brackets.*

Ion transport and binding, especially for the ferrous iron, were also the universal responding processes in *T. eurythermalis* A501. Ferredoxin was particularly important in the redox balance of *Thermococcales*, which is utilized as the only electron acceptor in glycolysis using the modified Embden–Meyerhof pathway (Nguyen et al., 2017). The active center of the ferredoxins is the iron-sulfur cluster ([FeS]), and the assembly proteins of the [FeS] cluster also responded to all tested stresses, which also supported the importance of ferrous iron in stress responses. Moreover, ferrous iron is essential for the active center in [NiFe] hydrogenases. In *T. eurythermalis* A501, [NiFe] hydrogenases include membrane-bound hydrogenase (MBH) and cytosolic sulfhydrogenase II (SHII). MBH contained at least one DEP responding to each tested stress, whereas SHII only contained DEPs to acid stress. The [NiFe] hydrogenase metallocenter assembly protein (Hyp), which provides the metallocenter for [NiFe] hydrogenase, was up-expressed under LpH, HpH, LS, and HS stresses but was down-expressed under LT stress and exhibited no response to HT or HP stresses. Finally, ferrous iron transporters, including ferrous iron transport protein FeoAB and ABC-type transport system FepBCD, contained at least one DEP responding to all tested stress except HpH stress (Supplementary Data S2 and Supplementary Figure S3).

Besides the above three enriched processes, the enzymes involved in biosynthesis pathways of canonical compatible solutes in hyperthermophiles, i.e., di-myo-inositol phosphate (DIP), trehalose, and cyclic 2,3-diphosphoglycerate (cDPG), were also detected in the A501 proteome as expected, as a universal response to multiple stresses (Table 2 and Supplementary Figure S3). Key enzymes in the synthesis pathway of DIP responded to heat, cold, and hyperosmotic stresses. Proteins involved in trehalose biosynthesis, transporters, and regulators responded to extreme salinity (LS and HS) and acid stresses (LpH), whereas cDPG synthase only responded to high pressure. In previous studies, DIP and trehalose have been reported to be accumulated under high salinity and heat stress conditions (Lamosa et al., 1998; Müller et al., 2005), similar to our observation. Although cDPG, thus far, has never been detected in *Thermococcales*, its synthetase was recently reported as a responding gene under high pressure stress in an obligate piezophilic *Thermococcales* strain *Pyrococcus yayanosii* CH1 (Michoud and Jebbar, 2016), which also matched our observation. These results indicate distinct roles of different compatible solutes, adopting an efficient strategy to protect the cell under multiple extreme conditions.

### Specific Dual-Stress Responding Processes

Beyond the above universal responding processes to three or more stresses, we also identified specific responses to two stresses. These dual-stress responding processes included (i) sodium-dependent energy conversion, which was differentially expressed under both high and low salinity stresses; (ii) membrane lipid biosynthesis, which was differentially expressed under cold and pressure stresses; and (iii) signal transduction, which was differentially expressed under heat and high-salinity stresses. Pairs of stresses ultimately impact the same cellular processes, suggesting that these pairs of stresses may have related effects on the cell to some extent.

#### Sodium-Dependent Energy Conversion Is the Limiting Factor Under Extreme Salinity

**FIGURE 4:**
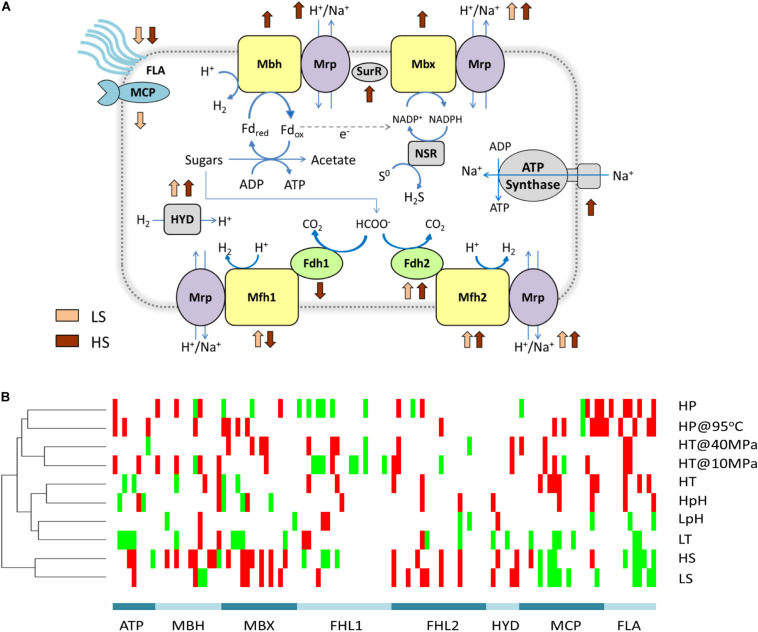
Energy metabolism responds to different environmental stresses. **(A)** Changes in energy metabolism responding to low and high salinity stresses. All genes of a complex responding to one stress in the same direction are shown in this graph. Membrane-bound complexes and oxidoreductases are in yellow. Sodium and proton antiporters are in purple. Formate dehydrogenase complexes are in green. Signal transduction and motility are shown in blue. Others are in gray. **(B)** Clustering heatmap of energy metabolism genes responding to different stresses. Significant DEPs were clustered in a heatmap. Up-expressed and down-expressed proteins and non-DEPs are presented in red, green, and white, respectively. MBH includes complexes of membrane-bound hydrogenase (Mbh) and Na^+^/H^+^ antiporter (Mrp). MBX includes complexes of membrane-bound oxidoreductase (Mbx) and Na^+^/H^+^ antiporter (Mrp). FHL1 includes complexes of formate dehydrogenase (Fdh1), membrane-bound hydrogenase (Mfh1), and Na^+^/H^+^ antiporter (Mrp). FHL2 includes complexes of formate dehydrogenase (Fdh2), membrane-bound hydrogenase (Mfh2), and Na^+^/H^+^ antiporter (Mrp). HYD: hydrogenase, including coenzyme F420 hydrogenase (Frh) and [NiFe] hydrogenase (Hyp). FLA, flagellum.

We also found that the energetic processes were especially important in extreme salinity: the GO term GO:0008137 was only enriched in both high- and low-salinity stresses among all the tested stresses (Figure 3 and Supplementary Data S3). Fifteen (of 17) significant DEPs in this GO term were the proteins in the membrane-bound complexes: membrane-bound hydrogenase (MBH), membrane-bound oxidoreductase (MBX), and two membrane-bound formate hydrogen lyase (FHL1 and FHL2). They were hypothesized to convert energy as a simple respiration chain with both the redox module and the Na^+^/H^+^ antiporter module (Mrp) in *Thermococcales* (Schut et al., 2013). Among these DEPs, the proteins in Mrp modules were up-expressed under both LS and HS stresses. Although the exact function of the above membrane-bound complexes remains elusive, they were believed to generate and regulate sodium and proton gradients for sodium-based A_1_A_0_ ATP synthase, which should be impacted by the sodium concentration under extreme salinity (Figure 4A).

To further explain the importance of energetic processes for extreme salinity, independent analysis of substance and energy metabolism was employed to provide more detailed information. The clustering analysis for DEPs of substance metabolism was similar to the clustering results for the total DEPs; however, the cluster for energy metabolism was different (Supplementary Figure S4). DEPs under both high and low concentrations of NaCl (HS and LS) were distinct from DEPs under all the other stresses (Figure 4B). This result indicated that the environmental concentration of sodium potentially played a key role in energy metabolism and might limit its growth under extreme salinity condition by disabling the energy conversion.

The sodium-dependent energy conversion might be the key limiting factor for NaCl adaptation. It could be a possible explanation for the narrow growth range of salinity in *T. eurythermalis* A501 (1–5% NaCl), which is in contrast to the wide ranges for pH, temperature, and pressure (Zhao et al., 2015). The narrow salinity ranges were also observed among other *Thermococcales* strains, such as *Thermococcus barophilus* (1–4% NaCl) (Marteinsson et al., 1999), *P. furiosus* (0.5–5% NaCl) (Fiala and Stetter, 1986), and *Palaeococcus ferrophilus* (2.0–7.3% NaCl) (Takai et al., 2000), as well as in other microorganisms that utilize sodium-based energy supply, such as *Methanothermobacter tenebrarum* (0–2% NaCl) (Nakamura et al., 2013) and *Methanosarcina acetivorans* (0.6–6% NaCl) (Sowers et al., 1984). Opposite to these sodium-dependent microorganisms, halophiles, which tolerate a wide range of environmental salinity, usually utilize the proton-based ATP synthase, such as *Halomarina oriensis* (0–3% NaCl) (Inoue et al., 2011), *Halalkalicoccus tibetensis* (8–30% NaCl) (Xue et al., 2005), and *Haloincolar saccharolyticus* (3–30% NaCl) (Zhilina et al., 1992) (Supplementary Figures S1, S5).

#### Membrane Lipid Biosynthesis Is Crucial to Cold and Pressure Stresses

The pathways related to membrane lipid synthesis were distinctly enriched under cold and pressure stresses. The phospholipid metabolic process (GO:0006644) was enriched under LT conditions, while the lipid biosynthetic/metabolic processes (GO:0008610, GO:0006629, and GO:0044255 with the same gene list for *T. eurythermalis* A501) were enriched under HP conditions (Figure 3 and Supplementary Data S3).

Similar to other *Thermococcales*, two important precursors are required in the synthesis of membrane lipids of *T. eurythermalis* A501: glycerol-1phosphate (Glyc1P), which provides the glycerophosphate backbone, and isopentenyl diphosphate (IPP), which provides the isoprenoid building blocks for the chain elongation (Koga and Morii, 2007; Jain et al., 2014; Villanueva et al., 2014).

Differentially expressed proteins responding to HP stress were related to the isoprenyl chain. Three key enzymes involved in mevalonate pathway and IPP production, i.e., hydroxymethylglutaryl-CoA synthase (TEU_00580), hydroxymethylglutaryl-CoA reductase (TEU_07265), and mevalonate kinase (TEU_05460), were all significantly down-expressed under HP, while the two key enzymes for chain elongation, isoprenyl diphosphate synthase (TEU_05485) and digeranyl-geranyl-glyceryl phosphate synthase (TEU_04265), were significantly up-expressed.

Compared with HP responses, the LT responding DEPs were related to phospholipid synthesis. The Glyc1P dehydrogenase (TEU_11035) producing Glyc1P, the first enzyme for the formation of polar lipids in archaea, were significantly up-expressed under LT stress. In addition, the archaetidylglycerol (AG) synthase (TEU_11545), producing AG from CDP-digeranyl-geranyl-glycerol phosphate, was significantly down-expressed under LT stress, while the inositol-1-phosphate synthase (TEU_00185), producing polar head for archaetidylinositol (AI), was significantly up-expressed. Both AG and AI belong to the archeol (C_20_). The opposing expression of these two enzymes suggested the potential shift from AG to AI in lipid composition under LT conditions.

The above results indicated that the HP stress might influence the synthesis of the isoprenyl chain for core lipids, while the LT stress might impact the synthesis of polar lipids. Overall, biosynthesis of membrane lipids plays an important role for both LT and HP stresses, which may be due to similar losses of membrane fluidity in cold and high pressure conditions (Oger and Jebbar, 2010).

#### Signal Transduction Is Crucial to Heat and Hypo-Osmotic Stresses

The proteins related to signal transduction were only enriched under the HT and LS stresses with the GO terms GO:0004871 and GO:0060089 (these two had the same gene list in *T. eurythermalis* A501) (Figure 3 and Supplementary Data S3). Signal transduction is one of the most general stress response processes, consistent with its roles in sensing pH, salinity, and toxins in both bacteria and archaea. The two-component signal transductions lead to both sense and bias movement of microorganisms in response to environmental stimuli (Boyd, 2015). In the gene list of the signal transduction of *T. eurythermalis* A501, chemotaxis proteins for signal sensing include CheA (TEU_11690), CheB (TEU_11685), CheW (TEU_11665), CheY (TEU_11685), and two methyl-accepting chemotaxis proteins (TEU_11670 and TEU_07885), while proteins for movement included three flagellins FlaB (TEU_11642, TEU_11645, and TEU_11635). Except for the non-significance of CheW, the above proteins were significantly down-expressed under LS stress. In contrast, except for the non-significance of CheW and one of the FlaBs, these proteins were significantly up-expressed under HT stress. These results indicated that the chemotaxis system in *T. eurythermalis* A501 might be negatively affected by the low concentration of NaCl but might be activated by higher temperature.

### Unique Responding Processes in Each Stress

The unique responding processes of each stress were also identified based on the GO enrichment results: carbohydrate catabolic process and cell division under cold stress, molybdopterin biosynthesis and metabolism under alkali stress, nuclease activity and DNA repair processes under HP stress, as well as the phosphorylation under HT stress (Figure 3 and Supplementary Data S4).

#### Cold Stress (LT)

The clustering heatmap and PCA plot presented that DEPs responding to cold stress was the most distinct from DEPs responding to other stresses (Figures 1B,C). During physiological experiments, the cultures under cold stress had the lowest specific growth rate and the lowest maximum growth yield; however, the number of down-expressed and unique DEPs under cold stress were the largest among all tested conditions (Table 1). These observations indicated a special feature responding to cold stress of such a hyperthermophilic strain.

Eight (of 10) DEPs involved in the carbohydrate catabolic process (GO:0016052) were significantly down-expressed under cold stress, including four energy-producing enzymes in glycolysis: ADP-dependent phosphofructokinase (PFK, TEU_11110), NAD(P)-dependent glyceraldehyde 3-phosphate dehydrogenase (GAPDH, TEU_08915), phosphoglycerate kinase (PGK, TEU_11225), and pyruvate kinase (PYK, TEU_09365). These down-expressed enzymes indicated a deficient glycolysis in *T. eurythermalis* A501 under cold stress.

Besides the carbohydrate metabolism, cell division (GO:0051301) was only enriched under cold stress. Four septum site-determining proteins MinDs (TEU_02560, TEU_07270, TEU_10955, and TEU_08700) contributing to cell division were down-expressed under cold stress. However, the energy-consuming enzymes involved in DNA replication, repair, and cell division were significantly up-expressed, including ATP-dependent DNA ligase (TEU_01440), GTP-binding protein EngB (TEU_04285), and cell division protein 48, which is an ATPase (TEU_11205). These results indicated a lack of energy for cell division under cold stress. Additionally, four (of nine) proteins in A_1_A_0_ ATP synthase (TEU_03105, TEU_03110, TEU_03115, and TEU_03120) were significantly down-expressed with 0. 22-, 0. 32-, 0. 58-, and 0.31-fold under cold stress, respectively (Supplementary Data S4), supporting the idea that the lack of energy occurred under cold stress. The above results indicated that deficient glycolysis and lack of energy for cell division occurred in *T. eurythermalis* A501 under cold stress, which might be an explanation for the lowest specific growth rate and lowest maximum growth yield among all tested conditions.

#### Acid Stress (LpH)

The oxidoreductase activity (GO:001662), acting on the aldehyde or oxo group of donors, with iron-sulfur protein as acceptor, was only enriched under acid stress. Six (of seven) DEPs were oxidoreductases involved in pyruvate and amino acid oxidation, including pyruvate oxidoreductase (POR), ketoisovalerate oxidoreductase (VOR), oxoglutarate oxidoreductase (OGOR), and indolepyruvate oxidoreductase (IOR). In *Thermococcales*, pyruvate plays two roles, as an acceptor for the amino acid-derived amino group via glutamate, forming alanine, and as an energy source to be oxidized to acetate through acetyl-CoA (Nohara et al., 2014). In amino acid oxidation, VOR, OGOR, and IOR oxidize 2-oxoglutarate derived from branched-chain amino acids, glutamate/glutamine, and aromatic amino acids, respectively. The branched-chain amino acid-specific VOR (TEU_01680) was up-expressed and was reported as an important energy-conserving enzyme in *T. kodakarensis* grown in the presence of S^0^. In contrast, the Glu/Gln-specific OGOR (TEU_08765) and aromatic amino acid-specific IOR (TEU_04855) were down-expressed. As an energy source, the key enzyme in pyruvate oxidation POR (TEU_01655 and TEU_01665) was up-expressed, which catalyzed the conversion of pyruvate to acetyl-CoA and transferred electrons to the energy conversion complexes MBH and MBX.

#### Alkali Stress (HpH)

Mo-molybdopterin cofactor biosynthesis and metabolism (GO:0032324, GO:0043545, GO:0006777, and GO:0019720) were only enriched under alkali stress. Molybdopterin is a cofactor consisting of molybdenum appended to GMP, CMP, AMP, or IMP (Johnson et al., 1993). Nine (of 13) DEPs involved in molybdopterin biosynthesis were up-expressed under alkali stress. Moreover, DEPs involved in purine-containing compound biosynthesis (TEU_09520, TEU_00750, and TEU_09080) and nucleoside transport (TEU_08830), which provided the components for molybdopterin, were also up-expressed (Supplementary Data S4). These results showed the importance and increased requirement of molybdopterin under alkali stress.

#### Pressure Stress (HP)

Differentially expressed proteins related to nuclease (GO:0016893) were down-expressed under HP stress, including DNA endonuclease (TEU_05000 and TEU_04710), tRNA nuclease (TEU_06440), and RNase (TEU_03540 and TEU_05555). In contrast, the DNA double-strand break repair-related enzyme (TEU_01120) and the chaperonin small heat shock protein (TEU_11270) in the stress response item (GO:0050896 and GO:0006950) were up-expressed. Compared with absent enrichment of structural and biosynthetic processes under HP, these findings indicated the importance of DNA repair systems and increased requirement of nucleic acid degradation. These results indicated that the HP stress may induce more DNA damages in this piezophilic strain than other stresses.

#### Heat Stress (HT)

For the responses to high temperature, which is one of the most interesting physiological features of this hyperthermophilic strain, we found that the transferring phosphorus-containing groups (GO:0016772) were the unique responding process that only enriched under heat stress but not under any other tested conditions (Supplementary Figure S3 and Supplementary Datas S3, S4).

Among all 18 proteins that were classified into this GO term, 12 were kinases, and 11 of these kinases were down-expressed, including the phosphoenolpyruvate carboxykinase (TEU_05830) and phosphoglycerate kinase (TEU_11225) in glycolysis, isopentenyl phosphate kinase (TEU_05465) and mevalonate kinase (TEU_05460) in lipid biosynthesis, as well as the other seven kinases using substrates as nucleoside diphosphate (TEU_09080), uridylate (TEU_04165), ribose (TEU_01625 and TEU_02660), and cofactors, i.e., NAD (TEU_02205), riboflavin (TEU_09890), and pantoate (TEU_01430). Among the total 12 kinases, only carbamate kinase (TEU_01535) was up-expressed, which is involved in the arginine biosynthesis (Supplementary Data S4). These results indicated a general negative effect on the expression of kinases under HT condition. Considering the close relationship between the phosphorylation function of kinases and cell signaling transduction, the up-expression of the chemotaxis proteins in signal transduction at HT indicated increased requirement in the signal transduction of the cell (details in Section “Signal Transduction Is Crucial to Heat and Hypo-Osmotic Stresses”); however, the down-expression of different types of kinases indicated that the negative effects of phosphorylation might be a limiting factor for the signaling transduction under HT stress.

## Discussion

In this study, we performed a global quantitative proteomic analysis under extreme temperatures, pH, HP, and salinity on a hyperthermophilic and piezophilic archaea strain, *T. eurythermalis* A501. The proteomic analysis with 79.8% genome coverage demonstrated that approximately 61.5% of the DEPs responded to multiple stresses, while only 1.5–7% of the total DEPs are uniquely responding to single stress. The GO functional enrichment of DEPs under each stress condition identified three levels of responses to various stresses: (i) universal responses to three stresses or more, (ii) specific dual-stress responses to two stresses, and (iii) unique responses for each tested stress. In total, DEPs involved in the enriched universal responding processes constitute 33.5% of the predicted protein-encoding genes in the complete genome of *T. eurythermalis* A501, while the DEPs involved in dual-stress or unique stress responding processes are less than 0.63%. These results indicated a cross-stress adaptation strategy in *T. eurythermalis* A501 to cope with the different types of the stresses.

The cross-stress adaptation behavior further indicated a high efficiency of multi-stress responses in the genome of *T. eurythermalis* A501. Utilizing universal responding processes to cope with multiple stresses should be a potential advantage for *T. eurythermalis* A501, which uses a merely 2.1-Mbp genome but grows over a wide range, a span of 50°C, 5 pH units, and 70 MPa, under multi-extreme conditions. The polyextremophile further reduced process complexity while inheriting relative shortage. Compared with the organisms using proton-driven energy-generating systems, the sodium-based archaea like *Thermococcales* show growth capability at wider ranges of pH and temperature but a narrower range for salinity (Supplementary Figure S5). This indicated that the sodium-based energy metabolism may contribute to the robust growth under extreme pH and temperature in these polyextremophiles like *Thermococcales*, but at the same time, it may limit the adaptation to the variation of sodium concentration and make the sodium one of the limiting factors by disabling the energy conversion.

The hyperthermophilic and piezophilic characteristic with the robust growth under multiple stresses make the *T. eurythermalis* A501 a potential ideal platform for the development of NGIB. The growth at high temperature provides possibilities for contamination-free bioprocessing, and the robust strain provides a stable platform for the changing of culture conditions. In this study, we highlighted the signaling transduction and the transferring phosphorus-containing groups as the specific and unique processes responding to high temperature in A501, which was rarely mentioned in previous studies of the high-temperature adaptation mechanism in hyperthermophiles and provided a new target for future studies.

Compared with temperature, pH, and salinity, the adaptation mechanism of the high HP is much less studied due to technical reasons (Zhang et al., 2015). Although most researches about pressure in biotechnology have been focused on the development of food processing methods, the application of elevated pressure in the range of 1–10 bar for bioprocesses is attracting more and more interests due to its great potential for enhancing process productivities and its lower cost in the long run compared to the use of oxygen-enriched air (Follonier et al., 2012). In this study, we demonstrated that the response to high pressure correlated with the responses to other stresses; positively correlated with alkali, high salinity, and heat stresses; and negatively correlated with acid and cold stresses (Figure 1A). The down-expression of the mevalonate pathway and the up-expression of the isoprenyl chain elongation under HP stress indicated the potential influences of HP on the length of isoprenyl chain in core lipids, which is similar to the observation in previous study (Cario et al., 2015a). Furthermore, we also highlighted the special requirements in nuclease activity and DNA repair processes under HP stress, which indicated the possibility of more DNA damages than other tested stresses, and may need more attention in future studies of the HP adaptation. These findings provided a novel perspective on piezophiles.

Hyperthermophiles are located in the roots of evolutionary trees (Stetter, 2006). The adaptation strategy of this hyperthermophilic archaea provides us a window to understand the important processes of early life. According to our observation, the responses to cold stress is the most distinct from the responses to all the other test stresses. Compared with the other tested stresses, *T. eurythermalis* A501 under cold stress has the lowest specific growth rate and lowest growth yield, but the largest number of down-expressed DEPs (Table 1). These phenomena indicated a deficiency in metabolism under cold stress. The significant down-expression of energy-producing enzymes in glycolysis and proteins in A_1_A_0_ ATP synthase revealed that the deficiency under cold stress could be related to the lack of energy. The down-expression of septum site-determining protein MinDs indicated the challenge of cell division under the energy-lacking condition of cold stress (Supplementary Data S2).

Besides the particularity of cold stress adaptation, we also observed the potential correlation among cold adaptation, acid adaptation, and antioxidation. Two essential antioxidative enzymes superoxide reductase (SOR) and the non-heme iron-containing protein rubrerythrin (Rdr) were up-expressed to 2.49- and 4.15-fold under cold stress in *T. eurythermalis* A501 and up-expressed to 3.82- and 2.15-fold under acid stress (Supplementary Data S2). In contrast, these two enzymes are not significantly differentially expressed at high-temperature stress. These results indicated similar increasing oxidation status under cold and acid conditions. The changing of the ancient earth, the decrease in temperature, and the increase in oxidation of the ocean were the major challenges for the ancient organisms with changing of the pH, and these problems must be solved in an efficient manner (Amend and McCollom, 2010). Our findings provided some hints that the cold stress adaptation in hyperthermophiles might be related to oxidation adaptation and has similar antioxidative behavior to the acid adaptation, which is worth further investigations to elucidate the adaptation mechanism of early life during the evolution.

## Data Availability Statement

The datasets generated in this study can be found in online repositories. The names of the repository/repositories and accession number(s) can be found in the article/ Supplementary Material.

## Author Contributions

WZ and XX designed this study. WZ, XM, XL, and YZ performed the experiments. WZ and XM contributed to data analysis. WZ and XX wrote the manuscript. HJ revised the manuscript. All authors reviewed the manuscript.

## Conflict of Interest

The authors declare that the research was conducted in the absence of any commercial or financial relationships that could be construed as a potential conflict of interest.

## Footnotes

1 http://www.geneontology.org/

## Supplementary Material

Ensure that any supplementary material is correctly published at this link: https://www.frontiersin.org/articles/10.3389/fmicb.2020.02081/full#supplementary-material

## Abbreviations

HP: high hydrostatic pressure, pressure stress, 85 ° C at 40 MPa vs. 85 ° C at 10 MPa
HP@95 ° C: pressure stress at high temperature, 40 MPa at 95 ° C vs. 10 MPa at 95 ° C
HpH: high pH
alkali stress: pH 8.8 vs. pH 7.0
HS: high salinity
hyperosmotic stress: 4.5% NaCl vs. 2.3% NaCl
HT: high temperature
heat stress: 95 ° C vs. 85 ° C
HT@10 MPa: heat stress at 10 MPa, 95 ° C at 10 MPa vs. 85 ° C at 10 MPa
HT@40 MPa: heat stress at 40 MPa, 95 ° C at 40 MPa vs. 85 ° C at 40 MPa
LpH: low pH
acid stress: pH 4.4 vs. pH 7.0
LS: low salinity
hypo-osmotic stress: 1.5% NaCl vs. 2.3% NaCl
LT: low temperature
cold stress: 65 ° C vs. 85 ° C.
