# Supplementary material for: Cross-Stress Adaptation in a Piezophilic and Hyperthermophilic Archaeon From Deep Sea Hydrothermal Vent
Source: Front Microbiol. 2020 Sep 10;11:2081. doi: 10.3389/fmicb.2020.02081 (PMC7511516; doi:10.3389/fmicb.2020.02081)
Supplement: Supplementary file 1 [file Data_Sheet_1.ZIP › Supplemental_material/Supplymental_Text.pdf]

Supplemental Data

Data S1. Universal and unique DEPs in *T. eurythermalis* A501 under the tested stresses. Universal, responded to more than two stresses. Unique, only responded to one stress. "*", *p*-value less than 0.05. Blank, *p*-value larger than 0.05, not significant. "---", not detected more than twice in the proteome. "HpH", pH 8.8 vs. pH 7.0. "LpH", pH 4.4 vs. pH 7.0. "LS", 1.5% NaCl vs. 2.3% NaCl. "HS", 4.5% NaCl vs. 2.3% NaCl. "LT", 65°C vs. 85°C. "HT", 95°C vs. 85°C. "HP", 85°C at 40 MPa vs. 85°C at 10 MPa.

Data S2. Protein expression in predicted active metabolic pathways of *T. eurythermalis* A501 in tested stresses. "*", *p*-value less than 0.05. Blank, *p*-value larger than 0.05, not significant. "---", not detected more than twice in the proteome. "HpH", pH 8.8 vs. pH 7.0. "LpH", pH 4.4 vs. pH 7.0. "LS", 1.5% NaCl vs. 2.3% NaCl. "HS", 4.5% NaCl vs. 2.3% NaCl. "LT", 65°C vs. 85°C. "HT", 95°C vs. 85°C. "HP", 85°C at 40 MPa vs. 85°C at 10 MPa. "HP@95°C", 40 MPa at 95°C vs. 10 MPa at 95°C. "HT@10MPa", 95°C at 10 MPa vs. 85°C at 10 MPa. "HT@40MPa", 95°C at 40 MPa vs. 85°C at 40 MPa.

Data S3. GO enrichment of *T. eurythermalis* A501 under each tested stress. Energetic processes responding to LS and HS, membrane lipids to LT and HP stresses and signal transduction to heat and low salinity stresses of *T. eurythermalis* A501.. "HpH", pH 8.8 vs. pH 7.0. "LpH", pH 4.4 vs. pH 7.0. "LS", 1.5% NaCl vs. 2.3% NaCl. "HS", 4.5% NaCl vs. 2.3% NaCl. "LT", 65°C vs. 85°C. "HT", 95°C vs. 85°C. "HP", 85°C at 40 MPa vs. 85°C at 10 MPa. **"**NS", no significant differential expression in that stress. "ND", not detected more than twice in the proteome. All the DEPs in the tables were the significant DEPs with folders higher than 1.2 or less than 0.8 and *p*-values less than 0.05.

Data S4. Unique response processes under each stress of *T. eurythermalis* A501 after GO enrichment. All the DEPs in this table have *p*-values less than 0.05. "HpH", pH 8.8 vs. pH 7.0. "LpH", pH 4.4 vs. pH 7.0. 2.3% NaCl. "LT", 65°C vs. 85°C. "HT", 95°C vs. 85°C. "HP", 85°C at 40 MPa vs. 85°C at 10 MPa.

Supplemental Figures

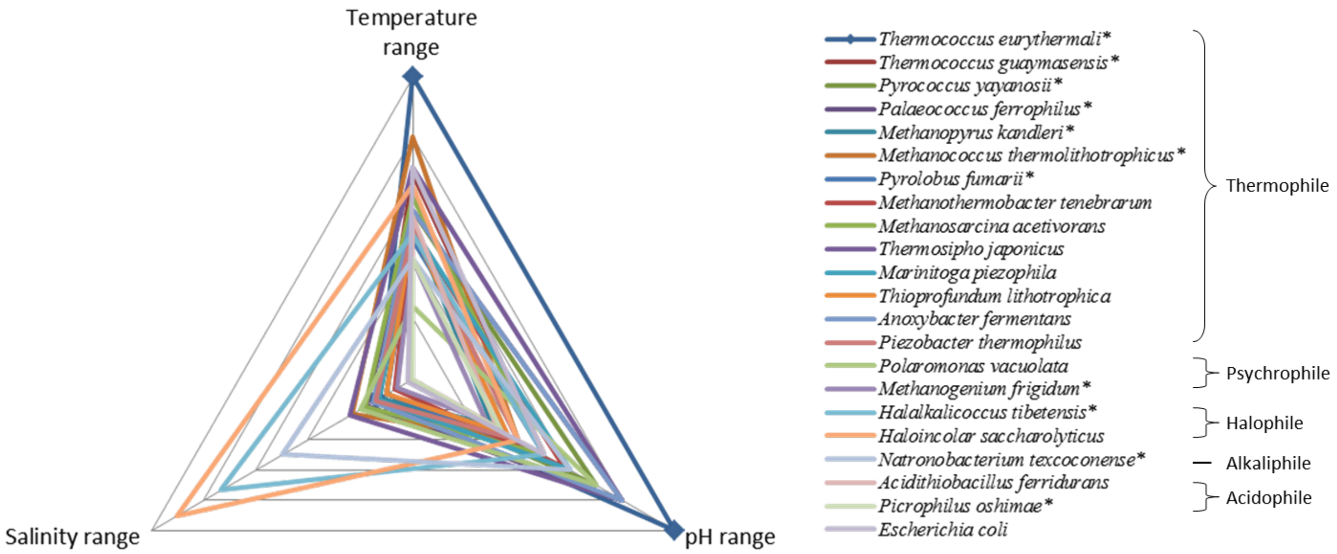

Fig. S1. Growth range of temperature, pH and salinity in different species. The radar map used normalized data with 50ºC for temperature range, 5 pH units for pH range and 30% NaCl for salinity range. The species name with * belongs to archaea, while bacteria with nothing. The growth range data of all the used species were from their type strains.

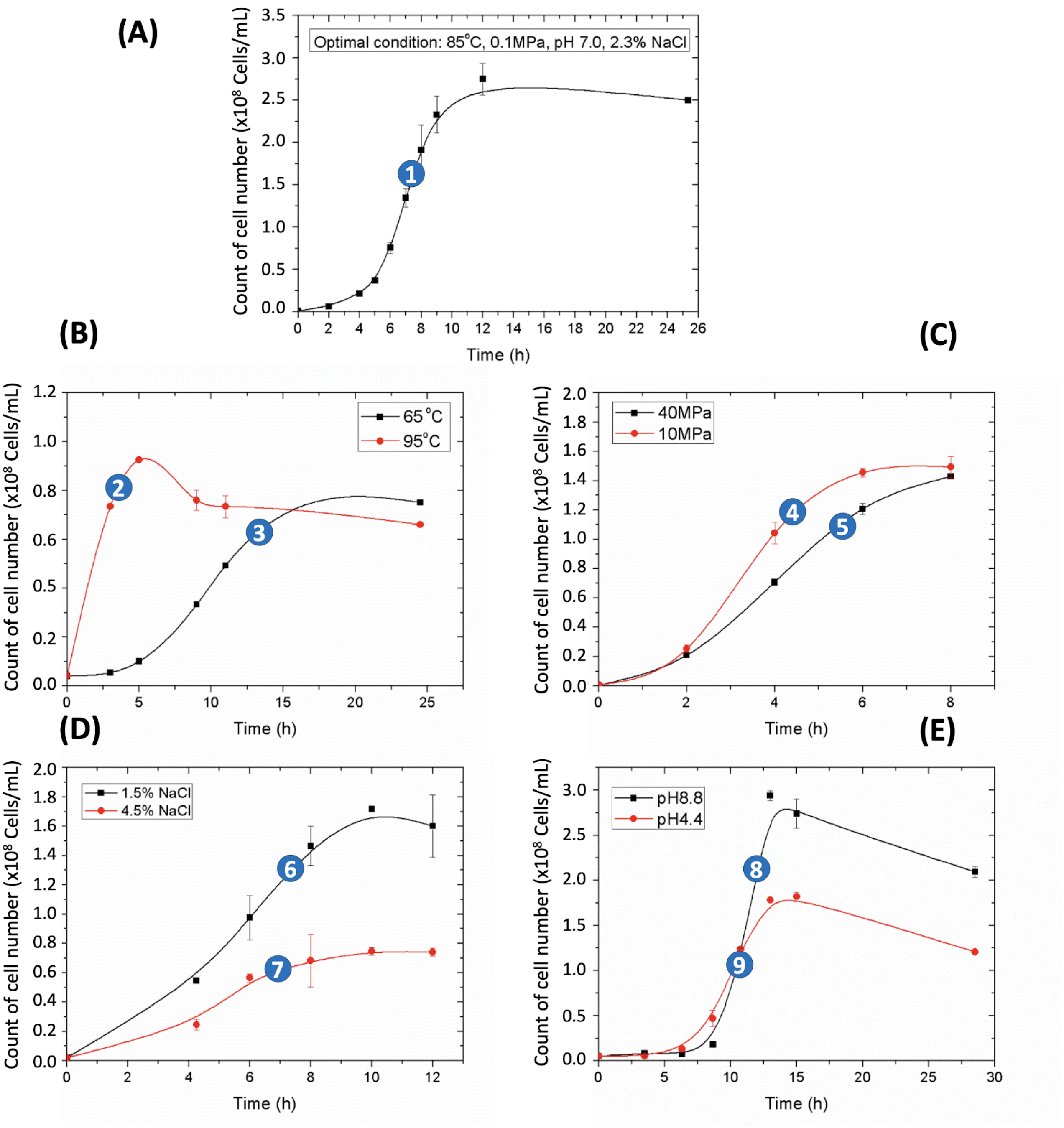

Fig. S2. Growth curves and sampling points of *T. eurythermalis* A501 under different stress culture conditions. (A) Optimal culture condition, sample 1 is the control. (B) Temperature stress, sample 2 at 95°C, sample 3 at 65°C. (C) Pressure stress, sample 4 at 10 MPa, sample 5 at 40 MPa. (D) Salinity stress, sample 6 at 1.5% NaCl, sample 7 at 4.5% NaCl. (E) pH stress, sample 8 at pH 8.8, sample 9 at pH 4.4. Cultures under each tested stress were performed in at least quadruplicate.

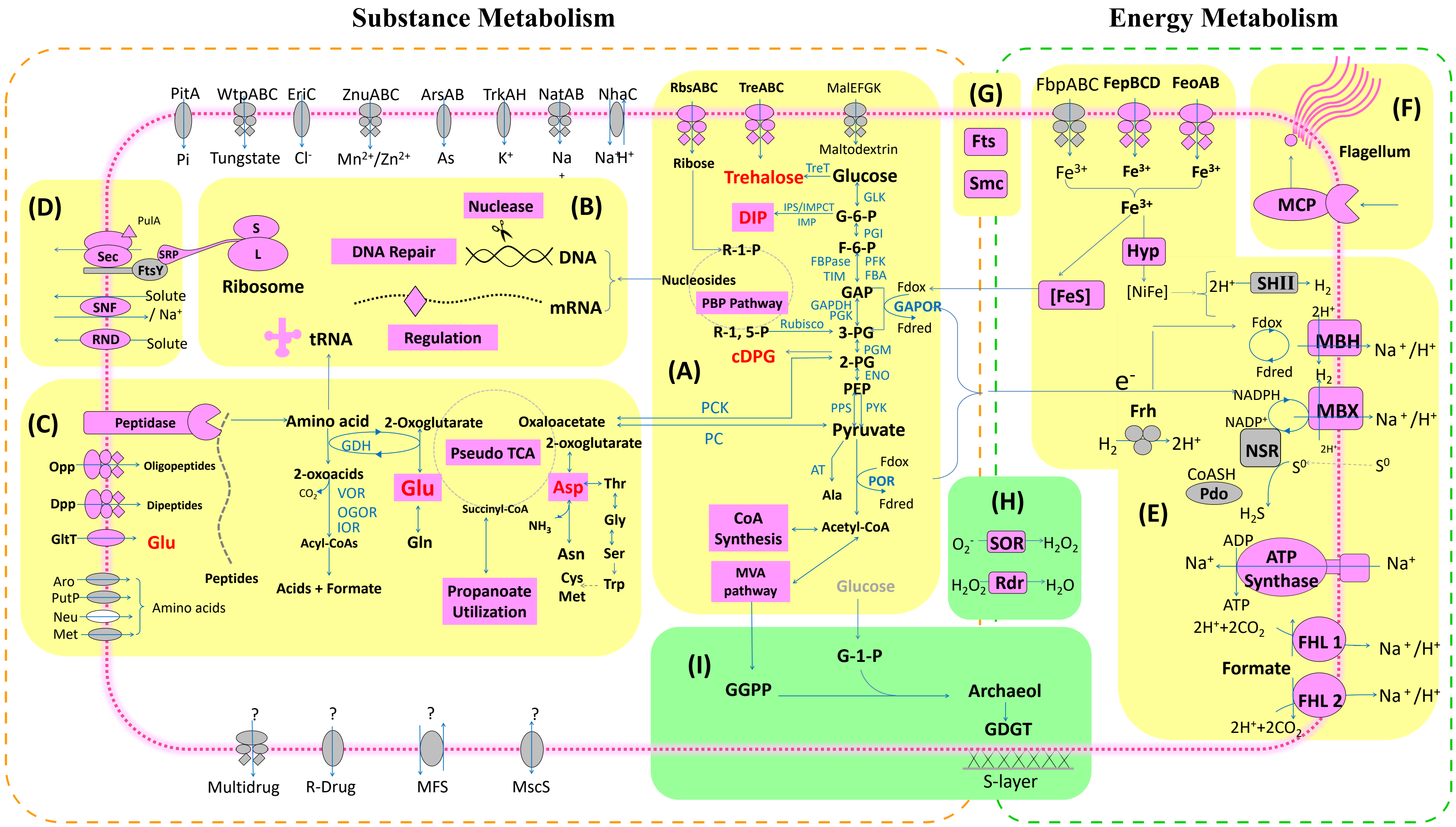

Substance Metabolism
Energy Metabolism
PitA
WtpABC
EriC
ZnuABC
ArsAB
TrkAH
NatAB
NhaC
Pi
Tungstate
Cl-
Mn2+/Zn2+
As
K+
Na+
Na+H+
RbsABC
TreABC
MalEFGK
Ribose
Maltodextrin
Trehalose
TreT
Glucose
GLK
DIP
IPS/IMPCT
IMP
G-6-P
PGI
R-1-P
F-6-P
FBPase
PFK
TIM
FBA
Nucleosides
PBP Pathway
GAP
GAPDH
PGK
Fdox
GAPOR
Fdred
Rubisco
R-1, 5-P
3-PG
cDPG
2-PG
PGM
ENO
(A)
PEP
PCK
PPS
PYK
PC
Pyruvate
AT
Ala
POR
CoA Synthesis
Acetyl-CoA
MVA pathway
Glucose
(G)
Fts
Smc
(H)
SOR
Rdr
(I)
G-1-P
GGPP
Archaeol
GDGT
S-layer
FbpABC
FepBCD
FeoAB
Fe3+
Hyp
[FeS]
[NiFe]
SHII
H2
2H+
MBH
Na+/H+
MBX
e-
Frh
NADPH
NADP+
NSR
S0
CoASH
Pdo
H2S
(E)
ADP
ATP
ATP Synthase
Na+
FHL 1
FHL 2
2H++2CO2
Formate
(F)
Flagellum
MCP
(D)
PulA
Sec
SRP
FtsY
SNF
Solute
/ Na+
RND
(B)
Nuclease
S
L
Ribosome
DNA Repair
DNA
mRNA
tRNA
Regulation
(C)
Peptidase
Amino acid
2-Oxoglutarate
Oxaloacetate
GDH
Pseudo TCA
2-oxoglutarate
2-oxoacids
CO2
VOR
OGOR
IOR
Glu
Asp
Thr
Gly
Ser
Trp
Succinyl-CoA
NH3
Asn
Cys
Met
Gln
Acyl-CoAs
Acids + Formate
Propanoate Utilization
Opp
Oligopeptides
Dpp
Dipeptides
GltT
Peptides
Aro
PutP
Neu
Met
Amino acids
Multidrug
R-Drug
MFS
MscS

Fig. S3. Proposed active metabolic pathway of stress adaptation of *T. eurythermalis* A501. (A) Carbohydrate metabolism. (B) Information processing. (C) Amino acid metabolism. (D) Secretion. (E) Energy conversion. (F) Motility. (G) Cell division. (H) Cytosolic antioxidation. (I) Membrane lipid biosynthesis. Universal pathways that responded to every tested stress have a yellow background, while specific processes responding to certain stresses have green backgrounds. Compatible solutes are in red and bold. Processes including proteins responding to at least three stress conditions are shown in pink. Proteins tested in the proteome but not differentially expressed are shown in grey.

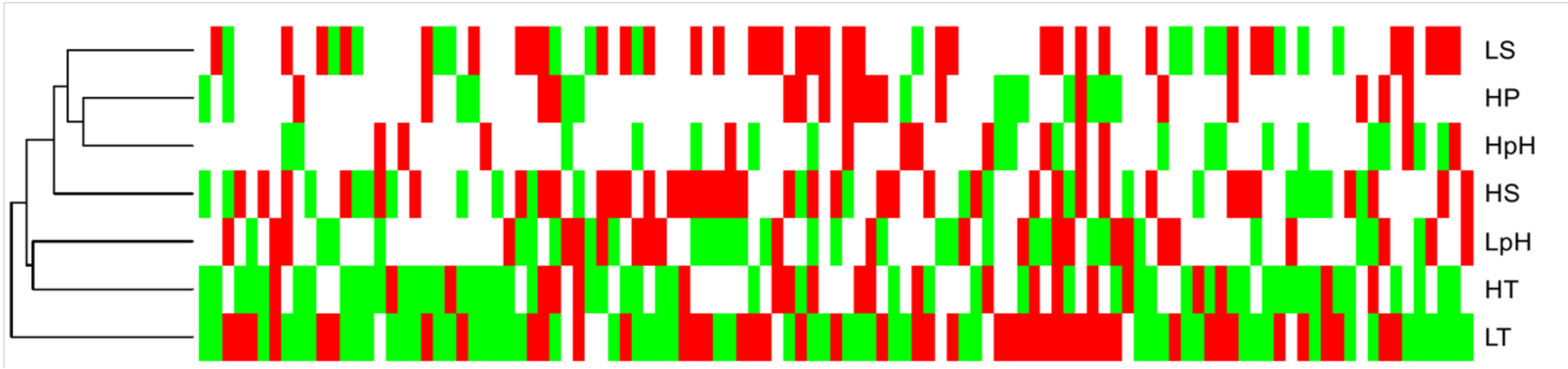

Fig. S4. DEPs in substance metabolism respond to different environmental stress. Up-regulated proteins are presented in red, down-regulated proteins are in green, and white indicates no significant differential expression.

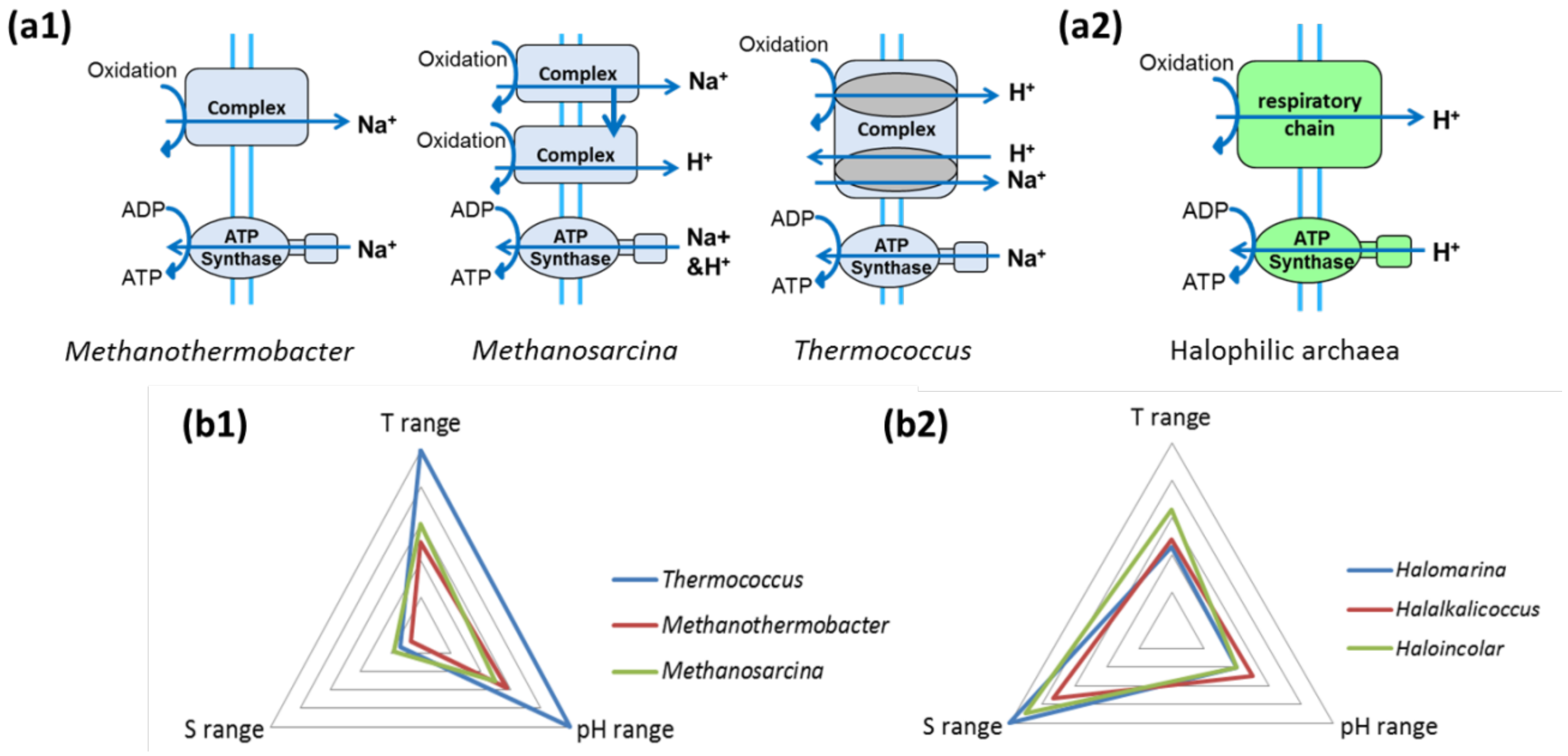

Fig. S5. Sodium- or proton-dependent ATP synthase related to growth range. (a1) Sodium-dependent ATP synthases are commonly present in hyperthermophilic archaea. (a2) Proton-dependent ATP synthases are used by typical halophilic archaea. (b1) Growth range of temperature (T), pH and salinity with concentration of NaCl (S). (b2) Growth range of typical halophilic archaea.

Supplemental Text

Amino acid requirements under different stresses:
The GO enrichment revealed that the biosynthesis and metabolism of amino acids responded to all tested stresses. In addition to the proteomic data, analysis of amino acid requirements showed that eight amino acids were essential for growth at optimal conditions, while the requirements of essential amino acids were varied with the stress conditions (**Supplemental Table S2**).

The essential amino acids were identified with a defined medium where amino acids were added as the only carbon source to displace yeast extract and tryptone in a TRM medium. The "lacking-one" regime, where one amino acid was excluded in the culture medium each time to make up 20 combinations of different amino acids, was applied, and the effect of the absence of each amino acid on cell growth was determined by cell counting under light microscope. To environmental stresses for cultivation were low temperature (75°C), high temperature (95°C), low pH (pH 4.5), high pH (pH 8.5), low salinity (1.5%), high salinity (4.5%), and HP (40 MPa), while the control was cultured at optimal condition (85°C, pH 7, 2.3% NaCl, 0.1 MPa). The inoculation was approximately 10^6 cells ml^{-1}. All the experiments were repeated at least with more than two replicates for each batch.

Aromatic amino acids were essential in every tested condition, including the optimal condition, and may be essential for growth under high temperature (Kannan and Vishveshwara, 2000). Branched amino acids were essential in every stress condition except the optimal condition, which may be due to the lack of energy supply under stresses; the branched chain in branched amino acids could be directly used as a carbon backbone. Asn and Gln were not essential in every tested condition, including the optimal condition. They may be independent from other amino acids and only used to conserve ammonia, while ammonia was supplied by *Thermococcales* Rich Medium (TRM) with $(NH_4)_2SO_4$ (0.5 g/L) (Zhao et al., 2015). Met was necessary in every tested condition except high pressure, while Cys was unnecessary in every tested condition. These results indicated that Met did not function in supplying sulfur. The highest number of amino acids were essential under low temperature stress, possibly because of the decrease in metabolic rate and the increase in antioxidative activity under low temperature. The requirement for Ala was only observed under low temperature, indicating deficiencies in metabolism and energy supply in cold stress. Ala could provide more pyruvate via a reverse reaction. Pyruvate is the most common intermediate product of glycolysis/gluconeogenesis, the rTCA cycle and pyruvate oxidation. Ser was needed only in low salt conditions, while Thr was essential in every tested condition, including the optimal condition. The extra hydroxyl of Ser could provide the hydrogen bond necessary to recover the dysfunction in low salt conditions. Proline was essential only under hyper- and hyposalinity.

Supplemental Table

Table S1. The essential amino acids for growth of *T. eurythermalis* A501 under different stresses. Branched: amino acids with branched chain; Positive charge: amino acids with a

positive charge; Polar: polar amino acids; Negative charge: amino acids with a negative charge; Aromatic: amino acids with an aromatic ring; (-OH): amino acids with a hydroxyl group; (S): amino acids containing sulfur. AA: amino acids; E: essential; Blank: non-essential.

**Classes**	**AA**	**Opt**	**LpH**	**HpH**	**LT**	**HT**	**LS**	**HS**	**HP**
	Gly								
	Ala				E				
**Branched**	Val		E	E	E	E	E	E	E
	Leu		E	E	E	E		E	E
	Ile		E	E		E			E
	Pro						E	E	
**Positive charge**	Lys		E	E	E		E	E	E
	Arg	E	E	E	E	E	E	E	E
	His	E	E	E	E	E	E	E	
**Polar**	Asn								
	Gln								
**Negative charge**	Asp	E	E	E	E				
	Glu								
**Aromatic**	Phe	E	E	E	E	E	E	E	E
	Tyr	E	E	E		E		E	E
	Trp	E	E	E	E		E	E	E
**(-OH)**	Thr	E	E	E	E	E	E	E	E
	Ser						E		
**(S)**	Met	E	E	E	E	E	E	E	
	Cys								

Supplemental References

Kannan, N., and Vishveshwara, S. (2000). Aromatic clusters: a determinant of thermal stability of thermophilic proteins. *Protein Eng.* 13, 753–761. doi:10.1093/protein/13.11.753.

Lamosa, P., Martins, L. O., Da Costa MS, and Santos, H. (1998). Effects of temperature, salinity, and medium composition on compatible solute accumulation by *Thermococcus spp. Appl. Environ. Microbiol.* 64, 3591–3598. Available at: https://www.ncbi.nlm.nih.gov/pubmed/9758772.

Michoud, G., and Jebbar, M. (2016). High hydrostatic pressure adaptive strategies in an obligate piezophile *Pyrococcus yayanosii*. *Scientific Reports* 6. doi:10.1038/srep27289.

Müller, V., Spanheimer, R., and Santos, H. (2005). Stress response by solute accumulation in archaea. *Curr. Opin. Microbiol.* 8, 729–736. doi:10.1016/j.mib.2005.10.011.

Zhao, W., Zeng, X., and Xiao, X. (2015). *Thermococcus eurythermalis sp. nov.*, a conditional piezophilic, hyperthermophilic archaeon with a wide temperature range for growth, isolated from an oil-immersed chimney in the Guaymas Basin. *Int. J. Syst. Evol. Micr.* 65, 30–35. doi:10.1099/ijs.0.067942-0.
